# HIV prevention cascades: A unifying framework to replicate the successes of treatment cascades

**DOI:** 10.1016/s2352-3018(18)30327-8

**Published:** 2019-01-01

**Authors:** Robin Schaefer, Simon Gregson, Elizabeth Fearon, Bernadette Hensen, Timothy B. Hallett, James R. Hargreaves

**Affiliations:** 1 Department of Infectious Disease Epidemiology, Imperial College London, Norfolk Place, London W2 1PG, United Kingdom (R Schaefer MPH, Prof S Gregson DPhil, Prof TB Hallett PhD); 2 Biomedical Research and Training Institute, Harare, Zimbabwe (Prof S Gregson DPhil); 3 Department of Public Health, Environments and Society, London School of Hygiene & Tropical Medicine, 15-17 Tavistock Place, London WC1H 9SH, United Kingdom (E Fearon PhD, Prof JR Hargreaves PhD); 4 Department of Clinical Research, London School of Hygiene & Tropical Medicine, Keppel Street, London WC1E 7HT, United Kingdom (B Hensen PhD); 5 Centre for Evaluation, London School of Hygiene & Tropical Medicine, 15-17 Tavistock Place, London WC1H 9SH, United Kingdom (Prof JR Hargreaves PhD)

## Abstract

Many countries are off track to meet targets for reducing new HIV infections. HIV prevention cascades have been proposed to assist in the implementation and monitoring of HIV prevention programmes by identifying gaps in the steps required for effective use of prevention methods, similar to HIV treatment cascades. However, lack of a unifying framework impedes widespread use of prevention cascades. Building on a series of consultations, we propose an HIV prevention cascade consisting of three key domains of motivation, access, and effective use in a priority population. This three-step cascade can be used for routine monitoring and advocacy, particularly by attaching 90-90-90-style targets. Further characterisation of reasons for gaps across motivation, access, or effective use allows for a comprehensive framework, guiding identification of relevant responses and platforms for interventions. Linking the prevention cascade, reasons for gaps, and interventions reconciles the different requirements of prevention cascades, providing a unifying framework.

## Strengthening HIV prevention through cascades

Global HIV incidence remains high and not only are many countries off track to meet international targets for reducing new infections,^1^ but the number of new infections increased in 50 countries between 2010 and 2017.^2^ Key populations, including men who have sex with men (MSM), sex workers, injecting drug users, and transgender people, and their sexual partners continue to experience particularly unacceptably high HIV incidence.^2^ Use of primary HIV prevention methods, including condoms, voluntary medical male circumcision (VMMC), pre-exposure prophylaxis (PrEP), remains limited,^3^ and funding for HIV prevention is often marginal compared to treatment.^4^ The 2018 UNAIDS Global AIDS Update speaks of a “prevention crisis”.^2^ The recently published report of the International AIDS Society (IAS)-Lancet Commission emphasises the possibility of a resurgence of the epidemic given the largest-ever generation of young people transitioning into adulthood.^5^ All of this underlines the need for novel approaches to strengthen implementation of HIV prevention programmes to increase the use of efficacious prevention methods.^6^

Measuring coverage and impact of HIV prevention programmes has been proven to be challenging, partly due to scarcity of data and lack of standard indicators.^2^ For treatment, the HIV treatment cascade, setting out the steps required for achieving viral suppression with antiretroviral therapy (ART), has provided a pragmatic and unifying framework for policy makers, programme planners, advocacy and civil society groups, and researchers,^7^ driving the impressive global scale-up of HIV treatment services.^2^ HIV prevention cascades have been proposed to similarly assist in the planning, monitoring, and improved delivery of HIV prevention programmes.^8,9^ The HIV prevention cascade aims to help programme planners, policymakers, and funders understand where there are gaps in primary prevention, by identifying and measuring the steps required for those at risk of infection to achieve effective use of prevention methods. The cascade can be used for routine monitoring of progress with use of prevention methods, and, in programme evaluation, can be a practical framework for organising data and prioritising interventions to improve impact. The cascade framework can serve to set meaningful prevention targets at international, national, and sub-national levels. These applications can improve accountability of HIV prevention programmes and those responsible for their implementation and can provide a basis for advocacy for prioritisation and increased funding.

To achieve this, the HIV prevention cascade should be adaptable across populations, prevention methods, and programmes. To be tractable for advocacy and regular monitoring, cascade formulations must be simple – so only the core steps and most essential determinants to achieve effective use of prevention methods should be included. These core steps need standardisation across methods so that comparisons can be made of progress with use of different prevention methods and across populations and over time and so that combination HIV prevention can be evaluated. Identifying specific gaps in prevention and selecting appropriate interventions to address these requires more comprehensive cascade formulations. Therefore, a unifying framework, which reconciles these requirements for simplicity and complexity, is needed for HIV prevention cascades.

Several formulations of HIV prevention cascades have been proposed. These differ in that they focus on particular prevention methods or populations (e.g. PrEP for MSM^10^), separate the perspectives of users and providers of prevention methods,^8^ or focus on interventions to address gaps identified in cascades rather than on the cascade for evaluating implementation of the prevention method.^9^ Comparisons across these cascades are difficult and lack of consensus impedes widespread use of prevention cascades. Most importantly, these cascades need further simplification and standardisation to fulfil requirements for advocacy and to facilitate monitoring and comparisons across programmes, populations, and over time. In this Viewpoint, we draw from and build on earlier work on HIV prevention cascades,^8,9^ a series of seminars and meetings (February 2017 – January 2018) with HIV researchers from a range of fields, and a consultation and workshop (31 July – 2 August 2017) involving local and international stakeholders in Harare, Zimbabwe, to address limitations in existing cascades and propose a unifying framework for HIV prevention cascades on which different applications with different requirements could be based.

## A unifying framework for HIV prevention cascades

**Figure 1:**
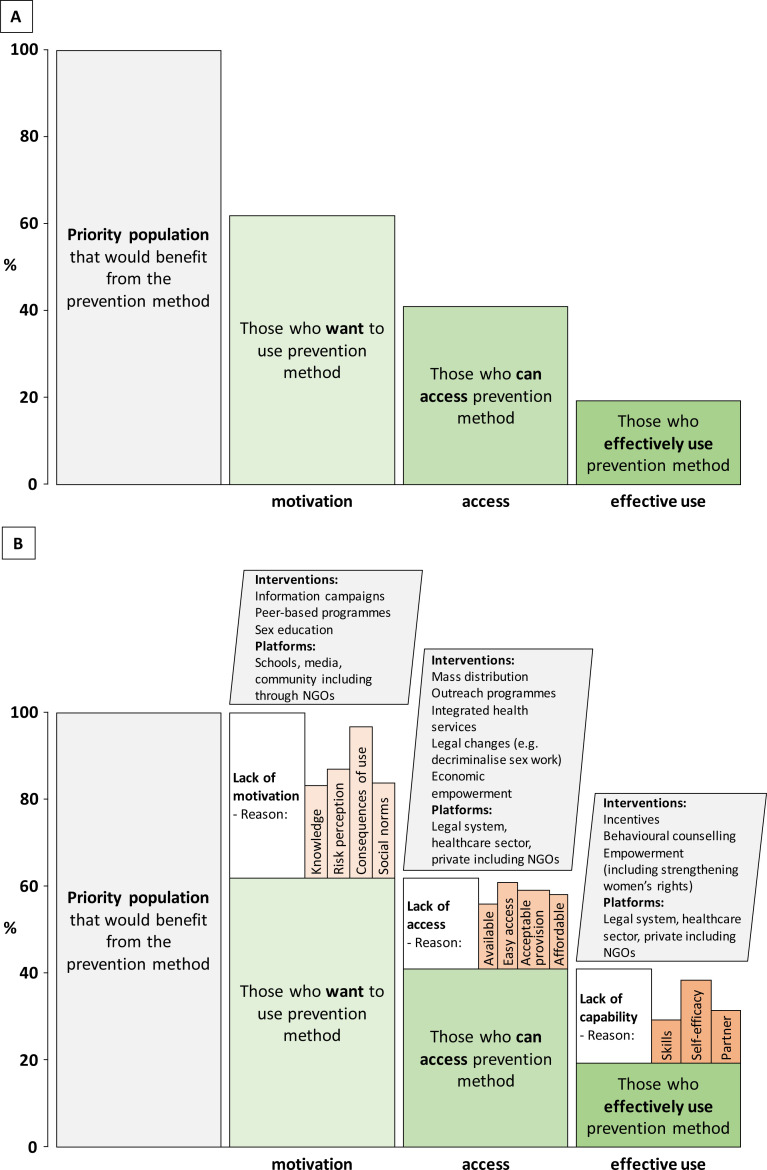
A generic and unifying HIV prevention cascade framework. **A:** The core steps of the cascade of motivation to use the prevention method, access to it, and effective use of it in a priority population that would benefit from use of the prevention method. **B:** The complete cascade that shows the gaps in the cascade across motivation, access, and effective use, and major reasons underlying these gaps. The displayed reasons do not represent an exhaustive list and, although some of these reasons are likely to be widely applicable, they may differ in relative importance between settings, populations, and prevention methods. The reasons provide links to interventions and platforms for interventions to improve motivation, access, and effective use in the priority population.

In seeking to integrate biomedical, behavioural, and structural approaches to HIV prevention, we recognise that HIV infection risk and use of prevention methods are fundamentally behavioural phenomena, facilitated or constrained by social and structural forces. To reflect this, we propose a generic HIV prevention cascade that identifies three key domains of motivation for using a prevention method, access to this method, and effective use in a priority population, reflecting capability to use and adhere to the prevention method (figure 1A). The priority population that could benefit from using a prevention method is made explicit as the identification of this is a task in itself.^1^ HIV prevention behaviour generally requires individual motivation to engage in these behaviours (although there may be exceptions, e.g. for condom use in sexual partnerships where one sexual partner may make a unilateral decision). Motivation – the cognitive processes that result in “wanting” to use a prevention method – is aligned with behavioural intent and resonates strongly with the so-called COM-B model of behaviour change in which behaviour is dependent on capability, opportunity, and motivation.^11^ This motivation may be wanting to use PrEP daily, condoms in particular sexual encounters, or use VMMC services, and is determined by multiple factors, including knowledge of the prevention method and its benefits, social norms, and perceptions about personal HIV infection risks. Motivation differs from the concept of demand in economics, which refers to consumer behaviour and covers preferences, agency, and affordability. However, motivation cannot translate into behaviour without provision of and access to HIV prevention methods where these are needed, which may be limited by high costs or inappropriate service provision. Motivation and access are linked but access can be considered irrelevant in the absence of motivation, so we suggest that motivation is the first step in the cascade. A step-wise cascade will be a more practical tool and we suggest that the order between motivation and access will not matter to programmatic decision making in most practical cases. Both motivation and access are requirements for effective use of prevention methods, but motivated individuals with access may still fail to use the method effectively if there are barriers to their underlying ability to adopt the behaviours in question, e.g. due to lack of skills, self-efficacy, or partner refusal. Therefore, effective use is the use required to achieve protection against HIV infection. In most cases (VMMC is a current exception), this protection will require adequate adherence over time during at-risk periods – a key challenge identified for condom and PrEP use.^12^

**Figure 2:**
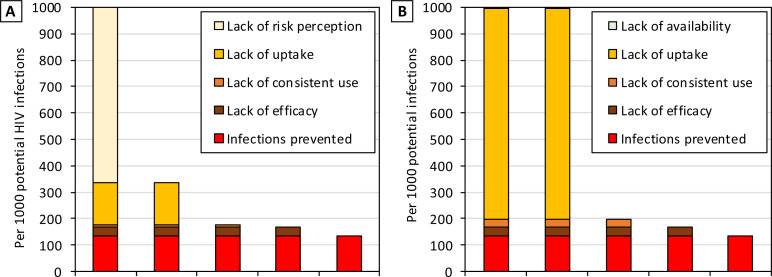
HIV prevention cascades following the models proposed by Garnett et al. for condom use among males in Manicaland, Zimbabwe, 2012-2013. The cascades show the number of HIV infections not prevented due to gaps in different cascade steps for males (aged 15-54) who reported sexual activity in the past two weeks and at least one sexual behaviour associated with increased risk for HIV infection (multiple, casual, or concurrent sexual partners), with HIV incidence estimated from the 2009-11 and 2012-13 Manicaland Study surveys (1.43 per 100 person-years). **A:** User-centric cascade with risk perception referring to reporting perceiving a risk for HIV infection in the future. **B:** Provider-centric cascade with availability referring to reporting knowledge of a place where condoms are available locally. Uptake was using a condom at least during one sexual encounter in the past two weeks. Consistent use was using a condom during every sex act in the past two weeks. Efficacy of 80% was assumed. Data on cascade steps were taken from the 2012-13 Manicaland Study survey. See supplementary material for details on data and methods.

Garnett and colleagues have proposed separate user- and provider-centric prevention cascades that differ in that the first step of the cascade is either perception of a personal risk of HIV infection (user-centric) or the availability of a prevention method (provider-centric) (figure 2).^8,13^ However, we believe that this separation of the user and provider perspectives is not desirable as a multitude of cascade models may limit the use of cascades and their comparability. Rather, we propose that cascades should be applied to and measured in priority groups that can benefit from HIV prevention methods, as is done in treatment cascades, since outcomes among these groups will ultimately determine impact and drive HIV prevention programming and policy.

Garnett et al. also proposed that the endpoint of the cascade should be the population-level impact of prevention use (HIV infections averted), allowing for quantification of infections not prevented due to gaps in the cascade. A sample application is shown in figure 2 for a population in Manicaland, eastern Zimbabwe (Manicaland Study) (see supplementary material for details on data and methods used to estimate the cascades). While HIV infections averted is an essential metric to measure the impact of HIV prevention interventions, we believe this should not be an integral function of the prevention cascade itself, just as estimating deaths averted by ART is not a part of the treatment cascade, as this would make prevention cascades difficult to operationalise. Although the link of use of HIV prevention methods with infections averted is not as close as that of treatment adherence and viral suppression with deaths averted, effective use – the final step in our cascade – is defined in a similar way, i.e. as the use required to achieve protection against HIV. This will vary between prevention methods and populations (e.g. PrEP for female sex workers vs. MSM^14–16^) but effective use can still be considered to be closely aligned with HIV infections averted for prevention methods with high efficacy when used consistently (e.g. condoms or PrEP). Including infections averted as the final step in the cascade could severely limit the use of the cascade concept as estimating infections averted is a complex exercise, requiring data that may not be feasible to collect at a local level and, to capture the true population impact of prevention interventions, estimating secondary infections prevented downstream in sexual networks. Estimating infections averted is particularly difficult for combination prevention. A presentation of the cascade with a scale of percentage (figure 1A) as opposed to ‘per 1000 HIV infections’ in the models by Garnett et al. (figure 2) is also a simpler and more easily understandable form of presentation, which is a major advantage of the treatment cascade. Nevertheless, our proposed cascade can be very usefully applied in mathematical models to estimate infections averted. This could be added as an extension to our proposed prevention cascade, which would further strengthen the advocacy applications of prevention cascades by illustrating potential impacts of improved prioritisation of prevention interventions. This may be particularly important for VMMC, where the link between effective use and infections averted is less clear given the incomplete efficacy of VMMC.^17^

Our proposed cascade with three core steps is generic in that it is not developed for a particular method or setting. Rather, it is designed to be used with any prevention method or combination of methods (such as PrEP or condoms) which makes it possible to compare the state of progress with use of different HIV prevention methods at a particular point in time (although the expected population-level impacts from effective use of different methods may not be directly comparable given different levels of efficacy) and also to compare HIV prevention use across populations and over time. It can be used for advocacy, routine monitoring, and strategic planning, and the simple three-step structure means that it can be used at all levels (e.g. country, district, and lower levels). Evidence-driven 90-90-90-style targets could be attached, further supporting the advocacy and strategic planning uses of the cascade.

**Figure 3:**
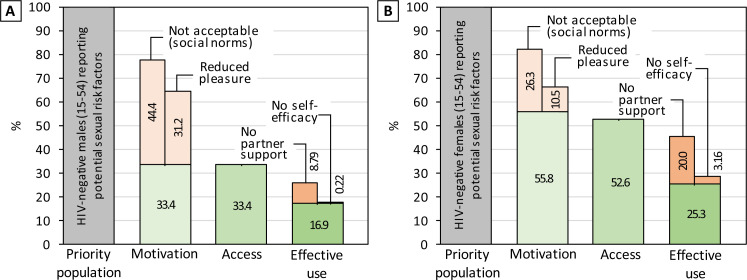
HIV prevention cascades following our proposed model for condom use among males and females in Manicaland, Zimbabwe, 2012-13. The priority populations were HIV-negative males **(A)** and females **(B)** (aged 15-54) who were reported sexual activity in the past two weeks and at least one sexual behaviour associated with increased risk for HIV infection (multiple, casual, or concurrent sexual partners). Individuals were classified as motivated if they reported perceiving a risk for HIV infection in the future. Among those classified as motivated, individuals were classified as having access if they reported knowing a place where condoms are available locally. Among those classified as having access, individuals were classified as effectively using condoms if they reported using a condom during every sex act in the past two weeks. Numbers are percentages of the priority population. See supplementary material for details on data and methods.

Figure 3 provides an example application of our proposed prevention cascade for condom use also using data from the Manicaland Study for comparisons with the Garnett et al. cascade (see supplementary material for details on data and methods). Among HIV-negative males and females who are considered a priority population as they reported sexual behaviours associated with increased risk of HIV infection (having multiple, casual, or concurrent sexual partners), the cascades illustrate gaps in motivation to use condoms, particularly among males, while the vast majority of individuals reported access to condoms. The cascades also illustrate that there were further gaps in effective use of condoms among motivated individuals with access, which was more pronounced among females.

Characterisation of cascade gaps across motivation, access, or effective use further increases the utility of the cascade as it shifts attention to where gaps in prevention lie, the reasons for these gaps, and, by identifying these reasons, possible ways to address the gaps, thus providing a comprehensive and action-oriented framework (figure 1B). This is particularly important for understanding reasons underlying lack of effective use as individual capabilities can determine use even where motivation and access do not represent strong barriers (for example, as is often the case for condoms where self-efficacy and factors relating to the partner may be particularly important^18^). Reasons for gaps across the three domains of the HIV prevention cascade may include factors specific to the individual but also social and structural factors; they may be applicable across different prevention methods or be specific to particular methods or populations.

Gaps in motivation to use a prevention method may relate to cognitive factors as motivation refers to a cognitive process, including lack of knowledge of a prevention method (e.g. of PrEP, which has only recently been introduced into many areas) or not perceiving a personal risk for HIV infection, but, equally, they could be due to social norms that limit the acceptability of the method (e.g. VMMC^19^). In the Manicaland example (figure 3), most respondents who were not motivated to use condoms reported negative social norms, and a large number of men reported reduced sexual pleasure as a negative consequence of condom use. Gaps in access may result from unavailability of the prevention method in the individual’s area, but, even where the method is available, structural barriers, including those relating to the legal system, or unacceptable aspects of service provision (e.g. lack of youth-friendly healthcare environments^20^) may limit accessibility. Effective use may also be limited by factors beyond the individual’s control; for instance, limited partner acceptability or exposure to gender-based violence.^21^ In Manicaland, for the gap in effective use of condoms among those motivated with access, lack of partner support was commonly reported, particularly by females, for whom the gap in effective use was more pronounced (figure 3).

Characterising reasons for gaps in the prevention cascade will help in identifying appropriate responses,^9^ which may include biomedical, behavioural, or structural interventions. For instance, interventions addressing gaps in motivation might target social norms surrounding use of prevention methods through comprehensive sex education.^22^ Interventions addressing access gaps could include outreach programmes to make prevention methods available and changes to the legal system like the decriminalisation of sex work^23^ or drug use^24^, removing fundamental barriers to access for these key populations. To address gaps in effective use, behavioural and couples counselling may improve skills, self-efficacy, and partner acceptability necessary for prevention use, while strengthening women’s rights may improve protection against gender-based violence (for instance, 36 countries in the world have no laws against domestic violence^25^). Prevention cascades, therefore, could help in identifying appropriate interventions by setting the targets – the gaps in prevention efforts that need to be addressed to increase use of prevention methods – but, in most situations, there will be a range of appropriate interventions to address the identified gaps, with the most appropriate choice depending on local circumstances. In other words, prevention cascades do not remove decision-making regarding intervention selection but rather narrow down area of focus for programme planners and policy makers. In the case of Manicaland (figure 3), gaps in motivation and effective use were identified, with social norms and partner approval being important reasons underlying these gaps, so community-based interventions may be appropriate to create a social environment conducive for HIV prevention, but the exact intervention is not determined by the cascade.

Learning from the treatment cascade, it is important to explicitly express how steps in the prevention cascade, the reasons for gaps in these steps, and the interventions that can address these reasons, are linked to avoid an excessive focus on a single step in a cascade (as some have argued has happened with the second ‘90’ of the treatment cascade^26^). These links underline the complexity of HIV prevention use and the interconnectedness of cascade steps, with interventions potentially having multiple impacts. For example, cash transfers to improve school attendance by adolescent girls may improve knowledge of HIV prevention methods and risk perception (i.e. increasing motivation), reduce poverty (increasing access), and reduce gender inequalities, improving negotiating skills, and economic dependence on sexual partners (increasing effective use).^27^ Finally, linking the gaps in the cascades to their determinants and, from these determinants, to interventions that address them provides a more comprehensive understanding of programme implementation and reconciles the requirements for simplicity and complexity, providing a unifying framework that supports all functions of the prevention cascade. While we argue that the three core domains of the cascade – motivation, access, and effective use – are applicable and key to nearly all types of HIV prevention methods and populations, the specific reasons underlying gaps in the cascade will often differ by context, populations, and prevention methods. Although we provide a broad range of reasons in figure 1B, these reasons are not an exhaustive list and will need local adaptation. This comprehensive HIV prevention cascade framework, therefore, guides not only identification of gaps in use of prevention methods but also the process that must be followed to fill these gaps, promoting a complete understanding of the needs of priority populations.

## Measurement of HIV prevention cascades

While the three-step core of the proposed HIV prevention cascade is simple, the steps themselves represent complex concepts. These could be measured with comprehensive indices, but it may only be feasible to collect such data in research studies. Instead, single measures for cascade steps will probably be needed for the cascade to be a pragmatic tool that can be implemented widely, measured using regularly collected data, and to ensure comparability across settings and over time. Motivation and access, as well as capability for effective use, are frequently neglected in commonly collected data such as UNAIDS monitoring indicators^28^ and even in HIV-focused research studies like the Manicaland Study. This is illustrated in figure 3 where we had to define motivation to use condoms as perceiving a personal risk for HIV infection, unrelated to condom use. Although risk perception may not necessarily translate into motivation for a specific behaviour,^29^ this measure had to be used as no data on “wanting” to use specific prevention methods were collected. Similarly, access had to be defined as knowing a place where condoms are available locally, but this does not necessarily imply that this is an accessible place for the individual. Nevertheless, single indicators for each cascade step for the main prevention methods could be measured in large-scale surveys like Demographic and Health Surveys or Population-Based HIV Impact Assessments.

While HIV prevention decisions and activities often occur outside of clinical settings, administrative data routinely collected in health systems could be useful for cascades for prevention methods like PrEP and VMMC. Data on motivation to use these methods could be collected during consultations, and uptake of these methods is already routinely collected. It may also be possible to create cascades combining surveys and routine programme data to gain a broader overview of the state of prevention use in a population.

These cascade measurements can be used for combination prevention, which is widely recommended as the most effective approach to HIV prevention programming.^30^ Combination prevention cascades reflect that individuals do not need to use all prevention methods and that use of one prevention method can influence use of another. Fearon et al. provide an example of a combination HIV prevention cascade for female sex workers in Zimbabwe.^31^ This cascade considered demand, supply, and effective use of condoms and PrEP separately but had the endpoint of being covered by either condoms or PrEP. This example can inform how the prevention cascade proposed here can be used for combination prevention: Motivation can be defined as wanting to use any one or several of a combination of prevention methods; access is the access to any of these prevention methods the individual wants to use; and effective use is the use of any of these prevention methods the individual wants to use and has access to. Data may be collected on motivation, access, and effective use for prevention methods individually, yet can easily be used for combination cascades that more accurately represent realities of use of HIV prevention methods in a population.

For some reasons underlying gaps in prevention cascades, improved measures may be needed (e.g. for HIV risk perception^32^). Standardised measurements may not always be feasible for individual reasons since these reasons will often vary between prevention methods and populations; however, standardisation is probably not essential at this level of detail since the emphasis will be on establishing what the reasons are in a particular context so that locally appropriate interventions can be identified.

## Moving forward

Applications of the HIV prevention cascade proposed here (and described in an informational video online^33^: https://youtu.be/7qQ5wm5tU_8) need to be piloted and demonstrated for different populations and using different data sources, including routine programme data – as has been done for treatment. Particularly, methods for measuring the three core domains of the cascade must be developed, validated and improved – e.g. by triangulating survey data with programme records and through qualitative studies – as poorly conceived or inappropriate indicators for cascade steps may create misleading cascades, resulting in inappropriate funding or programming decisions. Reorganising existing data, future data collection, and general thinking about prevention using the unifying HIV prevention cascade can improve implementation of programmes by identifying gaps in motivation, access, and effective use, and by providing new momentum to prevention through advocacy. The framework can be applied at all levels but guidelines are needed on how to incorporate it into national and international programmes, including targets for the major cascade steps. The HIV treatment cascade has shown how a unifying framework can become a driving force for programmes and policy nationally and internationally. A unifying HIV prevention cascade, when promoted, implemented, and evaluated, has similar potential to boost prevention efforts, curbing the spread of HIV in the many populations and areas in which HIV incidence remains unacceptably high and averting a potential resurgence of the epidemic.
